# Conformer-specific polar cycloaddition of dibromobutadiene with trapped propene ions

**DOI:** 10.1038/s41467-021-26309-5

**Published:** 2021-10-18

**Authors:** Ardita Kilaj, Jia Wang, Patrik Straňák, Max Schwilk, Uxía Rivero, Lei Xu, O. Anatole von Lilienfeld, Jochen Küpper, Stefan Willitsch

**Affiliations:** 1 Department of Chemistry, University of Basel, Klingelbergstrasse 80, 4056 Basel, Switzerland; 2 Center for Free-Electron Laser Science, Deutsches Elektronen-Synchrotron DESY, Notkestrasse 85, 22607 Hamburg, Germany; 3 Faculty of Physics, University of Vienna, 1090 Vienna, Austria; 4 Department of Physics, Universität Hamburg, Luruper Chaussee 149, 22761 Hamburg, Germany; 5 Department of Chemistry, Universität Hamburg, Martin-Luther-King-Platz 6, 20146 Hamburg, Germany; 6 Center for Ultrafast Imaging, Universität Hamburg, Luruper Chaussee 149, 22761 Hamburg, Germany

**Keywords:** Chemical physics, Reaction kinetics and dynamics, Chemical physics

## Abstract

Diels–Alder cycloadditions are efficient routes for the synthesis of cyclic organic compounds. There has been a long-standing discussion whether these reactions proceed via stepwise or concerted mechanisms. Here, we adopt an experimental approach to explore the mechanism of the model polar cycloaddition of 2,3-dibromo-1,3-butadiene with propene ions by probing its conformational specificities in the entrance channel under single-collision conditions in the gas phase. Combining a conformationally controlled molecular beam with trapped ions, we find that both conformers of the diene, *gauche* and *s-trans*, are reactive with capture-limited reaction rates. Aided by quantum-chemical and quantum-capture calculations, this finding is rationalised by a simultaneous competition of concerted and stepwise reaction pathways, revealing an interesting mechanistic borderline case.

## Abstract (web summary)

Identifying a concerted or stepwise mechanism in Diels–Alder reactions is experimentally challenging. Here the authors demonstrate the coexistence of both mechanisms in the reaction of 2,3-dibromobuta-1,3-diene with propene ions, using a conformationally controlled molecular beam reacting with trapped ions and ab initio computations

## Introduction

It has been almost a century since Otto Diels and Kurt Alder described the cyclisation reaction that is now widely known as the Diels–Alder (DA) cycloaddition^1^. In this reaction, a conjugated diene and an alkene, the dienophile, react to form a cyclohexene compound. Since its discovery in the 1920s, it has become one of the key reactions in synthetic organic chemistry for generating cyclic products^2^. Among others, it has been useful in the synthesis of many natural products such as the steroid hormone cortisone^3^, reserpine, a drug for treating high blood pressure^4^ or the antibiotic tetracycline^5^.

The ‘canonical’ mechanism of the DA cycloaddition assumes a concerted reaction proceeding via a single transition state in which bond formation and bond breaking occur synchronously^6–12^. In this case, the transition state is presumed to be stabilised by Hückel aromaticity involving [4 + 2]*π* electrons that renders the concerted mechanism energetically more favourable than the other limiting scenario, a stepwise process with a diradical intermediate^6,7^. The concerted mechanism explains why DA reactions often afford high stereo- and regioselectivity in the formation of the cycloadduct^6^. While often valid for symmetric systems, this picture breaks down in highly asymmetric systems in which the reaction becomes asynchronous to the extent that a stepwise mechanism is preferred^13,14^. In particular, this is the case for polar DA cycloadditions^15–17^ in which one of the reactants is charged. In the case of the [4 + 1^+^] polar cycloaddition, the removal of one electron from the dienophile leads to a radical cationic reaction in which the concerted transition state cannot be stabilised by Hückel aromaticity^7,18^. Owing to the practical importance of the DA reaction as well as its historical role in establishing quantum concepts in chemistry^19^, its mechanism has been the subject of numerous experimental and theoretical investigations over the past few decades and is still a subject of debate today^6–8,13,14,20,21^.

Experimental evidence for the mechanism of DA reactions has traditionally been obtained from kinetic isotope-substitution studies and from examinations of the regio- and stereospecificity of the reaction^6,22,23^. While the former method provides indirect evidence relying on the mechanistic interpretation of kinetic data, the latter only allows firm conclusions in the case of stepwise processes that are not stereoselective. A more direct and general route for the elucidation of the reaction mechanism can be provided by studies of the entrance channel of the reaction, in particular its conformational specificity. The concerted mechanism imposes that the reaction proceeds exclusively from the *s-cis* (or possibly *gauche*) conformer of the diene and not from the *s-trans* conformer, while a stepwise process would also enable the *s-trans* species to participate in the formation of the cycloadduct. Thus, information about the details of the reaction mechanism could be obtained from measuring the reaction rates of the individual conformers of the diene. However, the experimental challenges in isolating individual molecular conformations and their tendency to interconvert under ambient conditions in solution have rendered this approach for probing the reaction mechanism elusive so far.

A promising route for the investigation of chemical reactions in a controlled environment has recently been established with experiments combining molecular beams^24,25^ with trapped and Coulomb-crystallised molecular ions in the gas phase^26–28^. While a pure gas-phase approach naturally cannot account for solvent effects that often play a significant role in solution, it enables the characterisation of salient elements of reaction mechanisms under well-defined conditions in the absence of perturbations and thus also contributes to the understanding of reactions in solvated environments. Here, molecular beams generated by supersonic expansions allow molecular vibrations and internal rotations to be cooled down to very low temperatures such that individual molecular conformations are preserved. Their combination with inhomogeneous electrostatic fields has enabled the spatial separation of different conformers as well as individual rotational states based on their different electric dipole moments^29–32^. Directing such a controlled molecular beam at a reaction target of trapped ions has enabled kinetic and mechanistic studies of individual conformers of 3-aminophenol with Ca^+^ ions^33,34^ as well as nuclear-spin-selected water molecules with diazenylium ions (N_2_H^+^)^35^. In a recent study, we reported the successful electrostatic separation of the conformers of 2,3-dibromobuta-1,3-diene in a molecular beam^36^. DBB exists in an apolar *s-trans* and a polar *gauche* conformation (the *s-cis* conformation constitutes a saddle point on the potential-energy surface (PES) of this molecule).

**Fig. 1:**
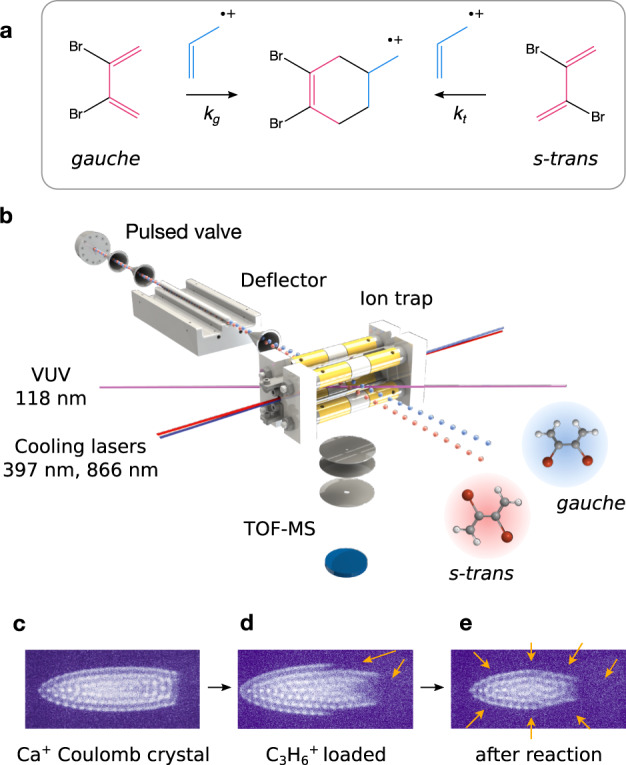
Overview of the experiment. **a** Scheme of the polar cycloaddition reaction between the *gauche* and *s-trans* conformers of 2,3-dibromobutadiene (DBB) with propene ions exhibiting reaction rate constants *k*_*g*_ and *k*_*t*_, respectively. The red and blue colours indicate the dienophile and the diene moieties, respectively. **b** Schematic of the experimental setup. The two conformers of DBB were separated by electrostatic deflection of a molecular beam and directed at an ion trap containing sympathetically cooled propene ions. Reaction products were measured by ion extraction into a time-of-flight mass spectrometer (TOF-MS). **c**–**e** Fluorescence images showing slices through laser-cooled Ca^+^ Coulomb crystals at different stages of the experiment: **c** shows the initial, pure Ca^+^ crystal, **d** shows an image after loading propene ions by vacuum-ultraviolet (VUV) photoionisation of propene and **e** is a typical image obtained after the reaction with DBB. Arrows indicate regions where ions heavier than Ca^+^ accumulate in the trap.

Here, we leverage these methods for the investigation of the mechanism and kinetics of a prototypical polar cycloaddition reaction. We study the [4 + 1^+^] cycloaddition of individual molecular conformations of DBB with propene ions (${{{{{{{{\rm{C}}}}}}}}}_{3}{{{{{{{{\rm{H}}}}}}}}}_{6}^{+}$) to form the 1,2-dibromo-4-methyl-cyclohexene radical cation (Fig. 1a). We find that both the *gauche* and the *s-trans* conformer of DBB react efficiently with propene ions. Quantum-chemical calculations of the PES of the system reveal a highly asynchronous reaction with a simultaneous competition of stepwise and concerted reaction pathways involving both conformers. The present system can thus be understood as an interesting borderline case that simultaneously incorporates both limiting DA mechanisms, illustrating the mechanistic complexity of DA reactions in ionic systems.

## Results

### Experimental setup

A schematic of the experimental setup is shown in Fig. 1b. A molecular beam of the rotationally and vibrationally cold neutral diene DBB in neon carrier gas was prepared by a pulsed supersonic expansion, see Methods and ref. ^36^. The collimated beam passed through an electrostatic deflector in which a strong electric-field gradient induced a spatially varying Stark-energy shift for polar molecules^24,36^. This resulted in a force acting on the polar *gauche*-DBB (dipole moment *μ*_gauche_ = 2.3 D^36^), which was thus vertically deflected from the beam axis in contrast to the apolar *s-trans* conformer (*μ*_trans_ = 0 D).

The molecular beam with the spatially separated conformers was directed at a linear-quadrupole radiofrequency ion trap (LQT) that contained a Coulomb crystal of laser-cooled Ca^+^ ions^28^. Fluorescence images of the Ca^+^ ions were recorded using a charge-coupled device camera, see Fig. 1c. The Ca^+^ Coulomb crystal served as a reservoir for the sympathetic cooling of propene ions as well as product ions formed during the reaction^26,28^.

The Coulomb crystal formed a stationary reaction target for the molecular beam. By vertically tilting the molecular-beam assembly, different parts of the DBB beam were overlapped with the ions in the trap entailing reactions with samples of different compositions of the *gauche* and *s-trans* conformers of DBB. This enabled the study of the influence of the DBB conformation on the cycloaddition kinetics. The formation of reaction products and the decay of reactant ions was measured by ejecting the ions from the trap into a time-of-flight mass spectrometer (TOF-MS) after a defined reaction time.

### Conformer separation of DBB

**Fig. 2:**
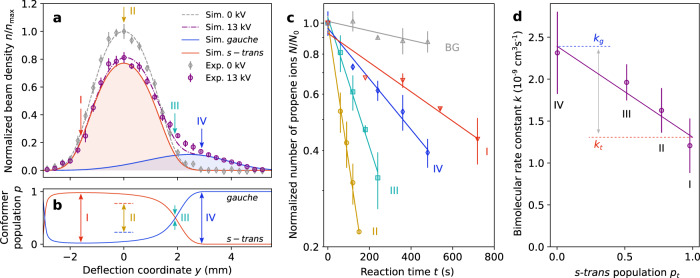
Conformer-specific reaction rate constants. **a** Measurement of the DBB beam-density profile along the deflection coordinate for deflector voltages of 0 and 13 kV^36^. The experimental data (symbols) are in good agreement with Monte–Carlo trajectory simulations (lines). At a deflector voltage of 13 kV, the two conformers were partially separated in the molecular beam. The roman numerals I–IV indicate the deflection coordinates at which reaction measurements were taken. **b** Populations of the conformers as a function of deflection coordinate in the molecular beam obtained from the trajectory simulations. **c** Reaction kinetics measured in terms of the normalised number of propene ions in the Coulomb crystal as a function of reaction time at the deflection coordinates I–IV marked in **a**, **b** and a background measurement (BG) without the molecular beam hitting the crystal. The exponential decay of the number of propene ions indicates a bimolecular reaction with pseudo-first-order kinetics. The data points have been normalised to the initial number of propene ions *N*_0_ for each measurement. **d** Bimolecular rate constants extracted from the pseudo-first-order rate constants as a function of the *s-trans*-conformer population. The line represents a linear fit to the data. Error bars correspond to one standard deviation of three independent measurements.

The density profile of the molecular beam of DBB was characterised along the vertical deflection axis. At specific tilt angles of the molecular beam, defining the deflection coordinate, the DBB molecules were ionised with vacuum-ultraviolet (VUV) radiation at a wavelength of 118 nm in the centre of the ion trap and subsequently ejected into the TOF-MS. Figure 2a shows normalised profiles of the beam density *n* obtained as a function of the deflection coordinate *y* measured with deflector voltages of 0 and 13 kV. The undeflected beam (grey symbols in Fig. 2a) contained a 1:3.3 mixture of the *gauche* and *s-trans* conformers of DBB, respectively, determined by the thermal populations in the room temperature reservoir from which the molecular beam emanated^36^. Separating the polar *gauche* from the apolar *s-trans* conformer using the deflector led to the appearance of a shoulder in the density profile towards larger deflection coordinates (purple symbols in Fig. 2a). The data are well reproduced by Monte–Carlo trajectory simulations of the molecular beam^36^ (lines in Fig. 2a) assuming a rotational temperature of 1 K and an independently measured beam velocity *v*_beam_ = 843(58) m/s^36^. These simulations were used in order to determine the populations of the conformers as a function of the deflection coordinate (see Fig. 2b) and to identify four positions, marked I–IV in Fig. 2a, b, that correspond to DBB samples with populations *p*_*t*_ of the *s-trans* conformer ranging from 1 to 0, respectively. Position II corresponds to the density maximum of the undeflected molecular beam (deflector voltage 0 kV) containing a thermal mixture of the conformers (*p*_*t*_ = 0.77). At positions I and IV, almost pure samples of *s-trans* and *gauche*-DBB were obtained, respectively. Position III corresponds to a mixture of practically equal contributions from both conformers.

### Reaction rate measurements

Reaction rate constants were determined following the procedure previously established in ref. ^35^. Exploiting the control over the conformational populations of DBB, reaction experiments were performed with the molecular beam at the deflection coordinates I–IV targeting the ion Coulomb crystal. The experimental sequence started by preparing a Coulomb crystal of about 10^3^ laser-cooled Ca^+^ ions in the LQT. Subsequently, propene ions were loaded into the ion trap by leaking propene gas into the vacuum chamber at a partial pressure of 3 × 10^−9^ mbar and photoionising propene molecules by VUV radiation. Due to their larger mass, the propene ions localised at the extremities of the Ca^+^ ion crystal as evidenced by a change of its shape (Fig. 1d). Note that no chemical reactions of neutral propene with the laser-cooled calcium ions were observed within the sensitivity limits of our experiment.

Reactions between propene ions and the DBB molecules were initiated by switching on the pulsed molecular beam. After a variable reaction time, the molecular beam was switched off. A typical Ca^+^ fluorescence image taken after reaction (Fig. 1e) shows a reduction of the number of Ca^+^ ions and a spatial rearrangement of the crystal due to trapped product ions. The ions were ejected into the TOF-MS to determine the number *N* of remaining propene ions. To discriminate between Ca^+^ (mass 40 u) and C_3_H_6_^+^ (mass 42 u), the TOF-MS was operated in a high-resolution mode within a limited mass range^37^.

Figure 2c shows measurements of normalised C_3_H_6_^+^ ion counts after reaction with DBB at the four molecular beam configurations I–IV as a function of reaction time. To account for the loss of propene ions due to collisions and reactions with background gas, background data sets were recorded for which the molecular beam was adjusted such that it did not impinge on the Coulomb crystal. An example of a background measurement is shown by the grey data points in Fig. 2c. Loss of propene ions due to collisions with neon from the molecular beam was found to be negligible in a control experiment with a pure beam of neon impacting on the crystal. All reaction measurements exhibited an exponential decay of the number of propene ions over time that implies a pseudo-first-order rate law for the bimolecular reaction, as expected for a constant DBB density replenished by the molecular beam.

The pseudo-first-order rate constants ${\tilde{k}}_{i}$ at beam positions *i* = I, …, IV were obtained from a linear fit to the logarithmic data (see Fig. 2c) and subtraction of the corresponding background rate constant. Bimolecular rate constants ${k}_{i}={\tilde{k}}_{i}/{n}_{i}$ were calculated using the DBB beam densities *n*_*i*_ determined from the beam-density profile shown in Fig. 2a and an independently measured absolute DBB density of *n*_avg_ = 3.9(4) × 10^6^ cm^−3^ at *y* = 0 and deflector voltage 13 kV (see Supplementary Note 1).

### Conformer-specific rate constants

Figure 2d shows the measured bimolecular rate constants *k* as a function of the *s-trans* population *p*_*t*_ obtained from the Monte–Carlo trajectory simulations, Fig. 2b. The bimolecular rate constant for the depletion of propene ions via the two reactions in Fig. 1a was modelled as a linear combination *k*_*i*_ = *p*_g,*i*_*k*_g_ + *p*_t,*i*_*k*_t_ of the rate constants *k*_g/t_ of the individual *gauche/s-trans*-conformers, respectively. The weighting factors *p*_g/t,*i*_ correspond to the respective conformer populations at molecular beam position *i*. The solid line in Fig. 2d represents a least-squares fit using the linear model for *k*_*i*_ that agrees with the experimental data within the error bars. The fit yields the bimolecular reaction rate constants *k*_g_ = 2.4(3) × 10^−9^ cm^3^s^−1^ for the *gauche* conformer and *k*_t_ = 1.3(2) × 10^−9^ cm^3^s^−1^ for the *s-trans* species. This implies that both conformers are reactive with *gauche-*DBB reacting faster than *s-trans*-DBB with a relative difference *r* = 2(*k*_g_ − *k*_t_)/(*k*_g_ + *k*_t_) = 0.6(1).

### Reaction products

**Fig. 3:**
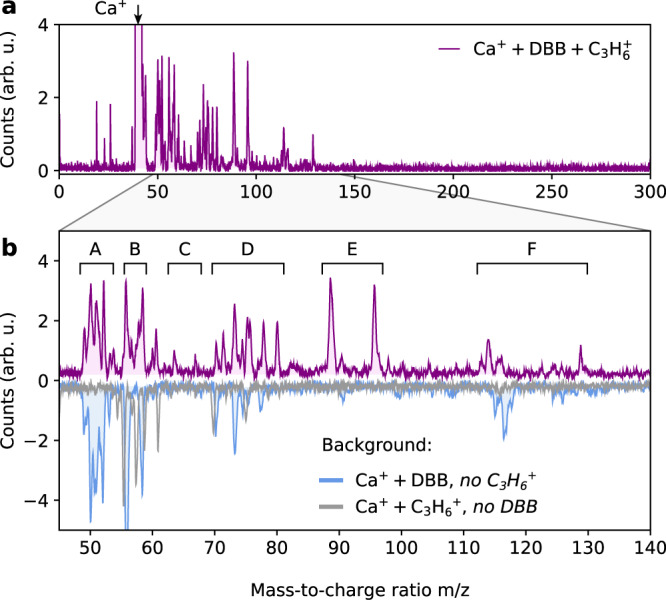
Mass spectra of reaction products. **a** Overview time-of-flight mass spectrum acquired after 2 min of reaction of DBB with propene ions. The spectrum shows a strong signal of Ca^+^ at 40 u and several product peaks. No signal could be detected at the expected mass of the cycloadduct at 252 and 256 u due to fragmentation. **b** Magnified view of the mass range showing the product signals (top trace) and comparison with control measurements in which either propene ions or DBB were absent from the experiment (lower inverted traces). The spectrum consists of several bands A–F corresponding to different fragments of the cycloadduct, see text for details.

To gain information about the products of this reaction, mass spectra of the trapped ions were recorded after 2 min reaction time. These experiments were performed with the molecular beam set to deflection-coordinate position II where the high beam density enabled fast data acquisition. The full mass spectrum, averaged over 50 experiments, is shown in Fig. 3a. Due to a combination of the strong exothermicity of the reaction (>60 kcal/mol) and the constant presence of near-infrared and near-ultraviolet laser light used to cool Ca^+^ (Methods), both of which promote fragmentation of the product ions, only fragments of the cycloadduct (mass *m* = 252, 256 u) could be observed. This is consistent with the behaviour observed in radical cation reactions of similarly sized systems^17^. The dominant peak due to Ca^+^ (40 u) was used to calibrate the mass scale of the spectra. An expanded view of the product-fragment spectrum is displayed in Fig. 3b and compared with control experiments either without propene ions in the trap (inverted blue trace) or without DBB in the molecular beam (inverted grey trace) under otherwise identical conditions. The TOF peaks can be grouped into several bands labelled with capital letters A–F (Supplementary Table 2). The bimodal structure of the peaks in the mass spectrum results from ions heavier than Ca^+^ forming extended shells around the Ca^+^ Coulomb crystal, so that they feel different extraction fields at different locations in the trap when accelerated into the TOF-MS. Therefore, the exact position and shape of the signals in the TOF-MS sensitively depends on the shapes and compositions of the multicomponent Coulomb crystals. The assignment of the signals in the TOF-MS to specific molecular compounds was based on detailed molecular dynamics simulations of the ejection process of the mixed species Coulomb crystals into the TOF-MS (see Supplementary Note 2).

The first two bands A and B were attributed to ${{{{{{{{\rm{C}}}}}}}}}_{4}{{{{{{{{\rm{H}}}}}}}}}_{{{{{{{{\rm{n}}}}}}}}}^{+}$ (*m* = 50–52 u) and CaO^+^ (*m* = 56 u), CaOH^+^ (*m* = 57 u), respectively, which were also present with the same intensities in the control experiments. This shows that ${{{{{{{{\rm{C}}}}}}}}}_{4}{{{{{{{{\rm{H}}}}}}}}}_{n}^{+}$ fragments were mainly formed by reactive collisions between Ca^+^ and DBB. Similarly, the band F was assigned to CaBr^+^ (*m* = 119 and 121 u) that also appeared in the control experiment in which C_3_H_6_^+^ was absent. CaO^+^ and CaOH^+^ were likely formed in a reaction of the Ca^+^ ions with residual H_2_O background gas in the vacuum chamber.

Fragments of the reaction product of DBB with the propene ions appeared as bands C, D and E. The strongest signal is observed for band E and can be attributed to a ${{{{{{{{\rm{C}}}}}}}}}_{7}{{{{{{{{\rm{H}}}}}}}}}_{9}^{+}$ (*m* = 93 u) fragment of the cycloadduct ${{{{{{{{\rm{C}}}}}}}}}_{7}{{{{{{{{\rm{H}}}}}}}}}_{10}{{{{{{{{\rm{Br}}}}}}}}}_{2}^{+}$, after removal of the two bromines and a hydrogen. Band D has a more complicated structure and is likely explained by the presence of ${{{{{{{{\rm{C}}}}}}}}}_{6}{{{{{{{{\rm{H}}}}}}}}}_{5}^{+}$ (*m* = 77 u) and ${{{{{{{{\rm{C}}}}}}}}}_{6}{{{{{{{{\rm{H}}}}}}}}}_{6}^{+}$ (*m* = 78 u). Finally, band C is consistent with the fragment ${{{{{{{{\rm{C}}}}}}}}}_{5}{{{{{{{{\rm{H}}}}}}}}}_{5}^{+}$ (*m* = 65 u) that may be formed by loss of a formal C_2_H_4_ unit from the ${{{{{{{{\rm{C}}}}}}}}}_{7}{{{{{{{{\rm{H}}}}}}}}}_{9}^{+}$ (*m* = 93 u) intermediate. The gradual decrease of signal intensity with decreasing mass from bands E to C suggests a stepwise fragmentation of the parent product ion. A more detailed discussion of the possible fragmentation pathways is given in Supplementary Note 2. In summary, the observation of all of these fragments is strong evidence that a DA cycloadduct is indeed formed in the reaction of DBB with propene ions.

## Discussion

Our experiments revealed a strong reactivity of propene ions with both conformers of DBB. Moreover, a pronounced enhancement of the reaction rate was observed with DBB in its *gauche* conformation. In order to understand the origin of this reactivity, quantum chemical calculations of the PES of this reaction were performed using density functional theory (DFT) (Methods). Since propene has a larger ionisation potential than DBB, the asymptote DBB + C_3_H_6_^+^ accessed in the experiments corresponds to the first electronically excited state of the ionic reaction system ≈8 kcal/mol above the ground-state asymptote. However, through long-range charge exchange via a conical intersection with the ground state early in the entrance channel, the reaction subsequently proceeds on the ground-state surface which asymptotically connects to DBB^+^ + C_3_H_6_.

**Fig. 4:**
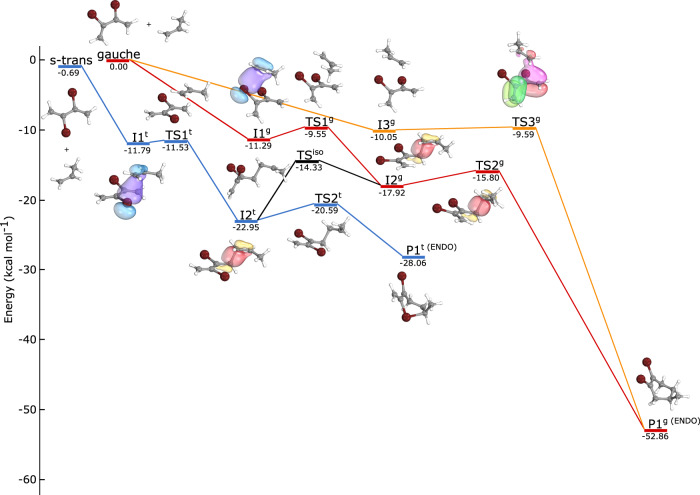
Potential-energy surface of the reaction. Simplified zero-point-corrected potential-energy profile of the electronic ground state of the [DBB-C3H6]+ system showing the most important structures along the minimum-energy paths for the polar cycloadditions of both conformers of DBB calculated using the M06-2X density functional and the def2-TZVPP basis set (Methods). A detailed PES containing additional conformational pathways and side-products is shown in Supplementary Fig. 5. Selected molecular orbitals that illustrate chemical bonds formed during the reaction are depicted for specific structures.

Figure 4 shows zero-point-corrected energies of important stationary points on the ground-state PES for reaction pathways of either conformer. A more detailed PES containing additional conformational pathways as well as pathways connecting to side-products is shown in Supplementary Fig. 5. For specific structures in Fig. 4, selected molecular orbitals are depicted that illustrate relevant chemical bonds formed during the reaction. For *gauche*-DBB, two pathways connecting to the endo and exo conformations of the cycloaddition product were found. One of these pathways proceeds via a single transition state (TS3^*g*^) and can therefore be classified as concerted^7^. However, this pathway is strongly asynchronous as TS3^*g*^ was found to be a loose transition state with a highly asymmetric structure exhibiting considerably different lengths of the newly formed C-C bonds (3.03 vs. 4.86 Å). By contrast, the second pathway is stepwise involving two separate bond-formation steps via the transition states TS1^*g*^ and TS2^*g*^ connected by intermediate I2^*g*^. Moreover, from I2^*g*^ a five-membered-ring structure P3^*g*^ can be formed that contains a hypervalent bromine atom and can rearrange to the DA adduct P2^*g*^ via TS5^*g*^ (see Supplementary Fig. 5).

For the *s-trans* conformer of DBB, a stepwise pathway connecting to the DA cycloadducts via intermediate I2^*t*^ was identified. From I2^*t*^, the *s-trans* path folds into the *gauche* path through a conformational rearrangement connecting to I2^*g*^ via TS^iso^. In other words, the *s-trans* form isomerises into the *gauche* conformer over the course of the reaction to connect to the DA products. This is not surprising as indeed, it is a geometrical requirement for ring formation. In addition, the isomeric five- and six-membered-ring structures P1^*t*^, P2^*t*^ and P3^*t*^ containing hypervalent bromine atoms are also accessible starting from I2^*t*^ (see Supplementary Fig. 5). These, however, are energetically not stable and will eventually isomerise to the DA adducts or fragment. Considering the substantial total energy release of more than 60 kcal/mol in the reaction, it can be expected that also the DA adduct will fragment under the present conditions, as was indeed observed experimentally.

Based on these theoretical calculations, it can be concluded that the polar cycloaddition of both conformers is energetically feasible in the present experiments, in line with the experimental findings. All barriers identified along the minimum-energy paths are submerged, i.e., their heights are below the energy of the reactants, thus rendering all identified pathways effectively barrierless.

**Fig. 5:**
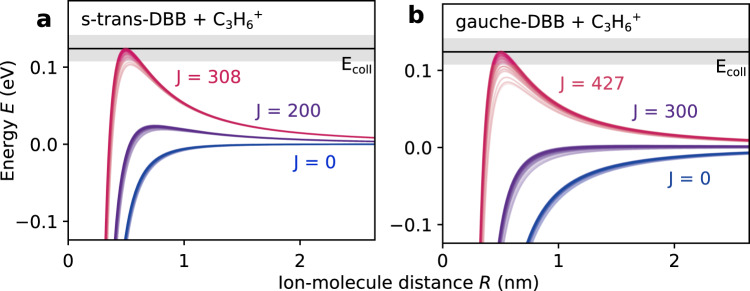
Adiabatic capture calculations. Rotationally adiabatic, centrifugally corrected ion-molecule interaction potentials for the reaction of **a** *s-trans*- and **b** *gauche*-DBB with propene ions for different values of the total collisional angular momentum quantum number *J*. Each value of *J* comprises a set of lines corresponding to all rotational states of DBB with rotational angular momentum quantum number *j* = 4 that corresponds to the largest population in the molecular beam. The collision energy *E*_coll_ is indicated by the black horizontal line and the grey-shaded areas represent its experimental uncertainty.

Motivated by these findings, the reaction kinetics were modelled using rotationally adiabatic quantum capture theory for barrierless ion-molecule reactions^38,39^ (see Supplementary Note 4). Within this framework, it is assumed that the short-range reaction occurs with unit probability and that the overall reaction rate is governed by the long-range interactions between the ionic and neutral collision partners and the centrifugal barrier. Figure 5a, b shows rotationally adiabatic, centrifugally corrected long-range interaction potentials for collisions of C_3_H_6_^+^ with *gauche*- and *s-trans*-DBB, respectively. They include the interaction of the ionic charge to the induced and permanent dipole moments of the neutral molecules. The three sets of curves correspond to different values of the total collisional angular momentum quantum number *J* that gives rise to the centrifugal energy barrier. The individual curves for each value of *J* correspond to all rotational quantum states of DBB with rotational angular momentum quantum number *j* = 4 that was populated the most in the molecular beam.

A unit reaction probability was assigned to any collision with $J \, < \, {J}_{\max }$^38,39^, where ${J}_{\max }$ is the maximum angular momentum quantum number for which the centrifugally corrected interaction energy does not exceed the experimental collision energy *E*_coll_ = 124(17) meV (black solid line in Fig. 5a, b). The plots show that the centrifugal barrier grows faster with *J* for *s-trans*-DBB than for *gauche*-DBB such that the maximum collisional angular momenta for a reactive encounter are *J*_max,*t*_ ≈ 308 and *J*_max,*g*_ ≈ 427, respectively, implying a larger cross-section proportional to ${J}_{\max }^{2}$^39^ for *gauche*-DBB. This is due to the attractive interaction of the ion with the dipole moment of *gauche*-DBB, which is not present for the apolar *s-trans* conformer. As a result, adiabatic capture theory predicts bimolecular rate constants of *k*_ac,*g*_ = 2.4 × 10^−9^ cm^3^s^−1^ for *gauche*-DBB and *k*_ac,*t*_ = 1.3 × 10^−9^ cm^3^s^−1^ for *s-trans*-DBB, in very good agreement with the measured values. The calculated relative difference of the reaction rate constants between the *gauche* and *s-trans* conformers is *r*_ac_ = 0.64 that also agrees well with the experimental value *r* = 0.6(1). The good agreement between the theoretical and experimental rate constants vindicates the mechanistic picture drawn from the quantum-chemical calculations above. It is interesting to note that although the magnitude of the rate constants is purely governed by long-range interactions, they reveal important details of the short-range mechanism, i.e., they clearly indicate the existence of a stepwise pathway for the *s-trans* conformer.

In conclusion, we have presented results on the chemical reactivity of individual molecular conformations in the prototypical polar cycloaddition reaction of 2,3-dibromobutadiene with propene ions. The current study represents an application of conformationally controlled molecular beams and ion-trapping methods for studies of the mechanism of organic name reactions under single-collision conditions in the gas phase. Our results indicate that both the *gauche* and the *s-trans* conformers of DBB react efficiently. This result is at variance with a purely concerted mechanism for the reaction in which only the *gauche* conformer is expected to react. The observed reactivity of both molecular conformations of the diene in conjunction with quantum-chemical calculations indicates the presence of stepwise reaction pathways for the cycloaddition in competition with a concerted process. We thus conclude that both limiting scenarios of the DA reaction mechanism are simultaneously active in the present system. The almost twofold larger rate constant for the *gauche* compared to the *s-trans* species was explained by attractive long-range ion-molecule interactions in very good quantitative agreement with an adiabatic capture model.

The present work demonstrates a platform for probing chemical kinetics with individual molecular conformations in model systems of fundamental organic name reactions under controlled conditions in the gas phase. In combination with mass-spectrometric tools, this capability opens up a wide range of opportunities for the precise investigation and validation of reaction mechanisms. Solvent effects, which may affect the mechanism in solution-phase and were not included in the present gas-phase study, could in principle be accounted for by using microsolvated reagents as has been demonstrated previously, for instance, in gas-phase mechanistic studies of S_N_2 reactions^40^.

## Methods

### Molecular beam

The molecular beam was generated from DBB vapour at room temperature seeded in neon carrier gas at 5 bar. The gas mixture was pulsed through a cantilever piezo valve (MassSpecpecD ACPV2, 150 *μ*m nozzle diameter) at a repetition rate of 200 Hz with a gas-pulse duration of 250 *μ*s measured at the position of the LQT. The speed of the resulting molecular beam was measured to be *v*_beam_ = 843(58) m/s^36^.

### Ion trap and TOF-MS

The LQT was operated at a peak-to-peak radiofrequency (RF) voltage of *V*_RF,pp_ = 800 V and a frequency Ω_RF_ = 2*π* × 3.304 MHz. Laser cooling of Ca^+^ ions was achieved using two continuous-wave laser beams at 397 and 866 nm generated by frequency-stabilised external-cavity diode lasers^26^. The Coulomb crystals were imaged by collecting the spatially resolved cooling-laser-induced fluorescence of the Ca^+^ ions with a microscope onto a camera (Fig. 1c–e).

The LQT was coupled to a TOF-MS orthogonal to the molecular-beam propagation axis for the mass and quantitative analysis of reactant and product ions^37^. For the measurement of overview mass spectra of the reaction products, a low-resolution operation mode was used for the extraction of ions into the TOF-MS by applying a 1 *μ*s long voltage pulse with a magnitude of 4.0 kV to the repeller electrodes. For the reaction rate measurements, the resolution was enhanced by applying an additional pulse of 4.0 kV, delayed by 0.45 *μ*s, to the extractor electrodes^37^. Ions were detected by a microchannel plate (Photonis) detector operating at a typical voltage of 2.3 kV placed at the end of the flight tube.

### Femtosecond laser and VUV ionisation

Ionisation of DBB molecules and Ca atoms was performed with pulses from a Ti:Sapphire femtosecond laser (CPA 2110, Clark-MXR, Inc.) at a wavelength of 775 nm and a pulse duration of 150 fs focused at the centre of the LQT. Ca atoms were ionised before loading into the ion trap in a standardised procedure. A constant size and composition of the crystals was verified by TOF-MS. Similarly, DBB from the molecular beam was ionised using femtosecond laser pulses to determine the beam density (see Supplementary Note 1). In addition, a pulsed VUV light source at 118 nm, focused down to a spot size of ≈100 *μ*m, was used for soft ionisation of the propene molecules and to measure the DBB deflection profile^36^.

### Quantum-chemical calculations

Quantum-chemical calculations of the PES were performed by spin-unrestricted DFT using the M06-2X functional^41^, which was evaluated for the treatment of DA reactions in refs. ^10,42^, with the def2-TZVPP basis set^43^ using the Gaussian 09 software package^44^. Stuttgart effective core potentials including scalar relativistic effects were used for ten inner-shell electrons of the bromine atoms on DBB^45^. In addition, the main stationary points were also found to be consistent with corresponding calculations based on the B3LYP functional^46,47^ with the exception of P1^*g*^ and P1^*t*^ that were not found to be local minima on the B3LYP level and directly converged to the P2^*g*^ and P2^*t*^ structures, respectively (see Supplementary Fig. 5). Moreover, the present DFT methodology was extensively validated against coupled-cluster and multireference methods with different basis sets in the related DBB + Ca^+^ reaction system, the details of which will be published elsewhere. Intrinsic reaction coordinates were computed to connect all transition states with intermediates shown in Fig. 4. The similar energy of TS6^*g*^ and P1^*g*^ arises from the addition of the zero-point energy and the very shallow nature of the TS. The single-point energies of the structures are listed in Supplementary Table 3. Bonding and wavefunction analyses were performed using intrinsic bond orbitals at the M06-2X/def2-TZVPP level of theory with the IboView programme package^48^. The spin contamination of the wavefunctions of the stationary points has been found to be small (see Supplementary Table 3), validating the application of the present single-reference level of theory.

## Supplementary information

Supplementary information

## Data Availability

The primary experimental data underlying the findings of this study as well as computed structures of stationary points on the PES have been deposited on the Zenodo repository under accession code 10.5281/zenodo.5525788.
