# Supplementary material for: Conformer-specific polar cycloaddition of dibromobutadiene with trapped propene ions
Source: Nat Commun. 2021 Oct 18;12:6047. doi: 10.1038/s41467-021-26309-5 (PMC8523519; doi:10.1038/s41467-021-26309-5)
Supplement: Supplementary file 1 — Supplementary information [file 41467_2021_26309_MOESM1_ESM.pdf]

Supplementary information for "Conformer-specific polar cycloaddition of dibromobutadiene with trapped propene ions"

Ardita Kilaj[1], Jia Wang[2*], Patrik Straňák[1*], Max Schwilk[3,1], Uxía Rivero[1], Lei Xu[1], O. Anatole von Lilienfeld[3,1], Jochen Küpper[2,4,5,6†], and Stefan Willitsch[1†]

[1] Department of Chemistry, University of Basel, Klingelbergstrasse 80, 4056 Basel, Switzerland
[2] Center for Free-Electron Laser Science, Deutsches Elektronen-Synchrotron DESY, Notkestrasse 85, 22607 Hamburg, Germany
[3] University of Vienna, Faculty of Physics, 1090 Vienna, Austria
[4] Department of Physics, Universität Hamburg, Luruper Chaussee 149, 22761 Hamburg, Germany
[5] Department of Chemistry, Universität Hamburg, Martin-Luther-King-Platz 6, 20146 Hamburg, Germany
[6] Center for Ultrafast Imaging, Universität Hamburg, Luruper Chaussee 149, 22761 Hamburg, Germany
∗ These authors contributed equally to the present work.
† Electronic mail: stefan.willitsch@unibas.ch, jochen.kuepper@cfel.de

Supplementary Note 1 Molecular beam density

The density of DBB molecules in the molecular beam, n_{DBB}, was determined in order to convert the measured pseudo-first-order rate constants into bimolecular rate constants. For this purpose, the sensitivity of the TOF-MS was first calibrated to be able to convert the integrated MCP signal into an absolute number of ions [1]. Using this information, the DBB density was determined from the dependence of the photoion yield on laser intensity in a strong-field multi-photon ionisation experiment [2, 3].

1.1 Calibration of the TOF-MS sensitivity

In order to calibrate the sensitivity of the TOF-MS, the TOF-MS signals generated by Coulomb crystals of a well-defined ion number were measured [1]. Fig. 1a shows exemplary fluorescence images of Ca^+ ion strings containing two to five ions. Counting the number of ions in each image was facilitated by integrating the fluorescence in the images along the vertical axis. The corresponding integrated fluorescence curves are shown below the images with the red line corresponding to a moving average over 11 points. Fig. 1b shows the correlation between the number of ions and the total integrated fluorescence counts for a selection of 28 Coulomb crystal images with up to 9 ions. Fitting the data with a linear function gives a slope of 0.65(6) counts per ion. This relation enabled the determination of the number of ions using the integrated fluorescence of any Ca^+ Coulomb crystal. For a larger set of Coulomb crystals, both fluorescence images as well as TOF-MS data were acquired. Fig. 1c shows the integrated Ca^+ TOF-MS signal as a function of ion number, determined from fluorescence counts. Fitting these data with a linear function gives a TOF-MS sensitivity of 0.136(14) Vns/ion, where the uncertainty also includes the uncertainty in the relation between ion number and fluorescence counts.

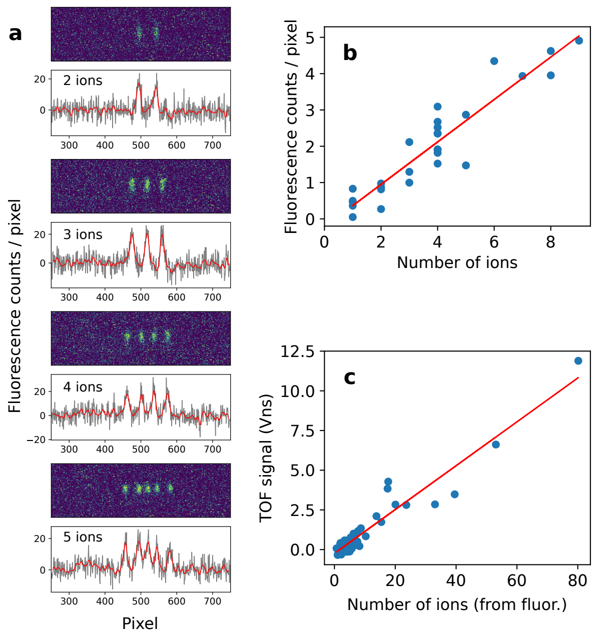

Supplementary Figure 1: **Calibration of the TOF-MS. a**, Fluorescence images of Ca^+ Coulomb crystals with 2 to 5 ions (top to bottom). The fluorescence integrated along the vertical image axis is shown below each image. The red line corresponds to a moving average of over 11 points. **b** Integrated Ca^+ fluorescence counts as a function ion number. The red line corresponds to a linear function fitted to the data. **c** Integrated Ca^+ TOF-MS signal as a function of ion number (determined from measured fluorescence counts based on the fit function in **b**. The red line corresponds to a linear function fitted to the data.

1.2 Determination of the density of the DBB molecular beam

The density of DBB molecules in the molecular beam was determined using ionisation yields in non-resonant strong-field ionisation [2, 3]. Here, a voltage of 13 kV was applied to the deflector and the molecular-beam apparatus was set to a deflection coordinate of $y = 0$ mm. The molecular beam was ionised by multi-photon ionisation using laser pulses with a duration of 150 fs at 775 nm. The total ion yield was measured as a function of the laser-pulse intensity, Fig. 2 and was found to scale logarithmically with laser intensity I for $I > 3.3 \times 10^{14}$ W/cm^2. This behaviour is expected for saturated multi-photon ionisation [2] and allows the extraction of the molecular-beam peak density n_{peak} from the slope of the line [3]

$$m := \frac{\mathrm{d}S}{\mathrm{d}\ln I/I_0} = 2\pi\alpha\sigma_r^2 d n_{\text{peak}} \tag{1}$$

where S is the ion count, I is the laser intensity, I_0 is the saturation intensity of the relevant molecular species, $\alpha = 0.57$ is the detection efficiency, $\sigma_r = 7.5$ μm is the $e^{-1/2}$ radius of the laser beam and $d = 2.5$ mm is the diameter of the molecular beam. We determined $m = 39(2)$ and $I_0 = 2.56(5) \times 10^{14}$ W/cm^2 from a linear fit of S as a function of $\ln(I)$ (Fig. 2). Using (1), a DBB density of $n_{\text{peak}} = 7.8(7) \times 10^7$ cm^{-3} was obtained. The uncertainty includes the error of the TOF-MS calibration. Because the molecular beam was pulsed with a repetition rate $f_{\text{rep}} = 200$ Hz and a pulse duration of $\tau_{\text{pulse}} = 250$ μs at the position of the LQT, the time-averaged density relevant for the reaction experiments was calculated to be $n_{\text{avg}} = n_{\text{peak}} f_{\text{rep}} \tau_{\text{pulse}} = 3.9(4) \times 10^6$ cm^{-3}.

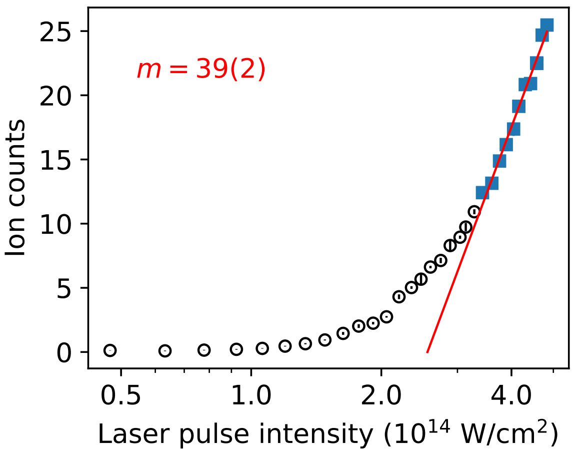

Supplementary Figure 2: **Calibration of the DBB beam density.** The ion yield from fs-laser photoionisation was measured as a function of the laser intensity. At intensities $I > 3.3 \times 10^{14}$ W/cm^2 (blue squares), the ion yield is well represented by a logarithmic dependence on the laser intensity (red line) from which the beam density can be determined (see text).

Supplementary Note 2 TOF mass spectra of reaction products

2.1 Molecular dynamics simulations

For an accurate assignment of the signals in the TOF mass spectra shown in Fig. 3 of the main text, molecular dynamics (MD) simulations of mixed species Coulomb crystals ejected into the TOF-MS were performed using the SIMION software [4]. In an ion Coulomb crystal, ions spatially arrange in shells according to their mass-to-charge ratio. While lighter ions accumulate closer to the centre, heavier ions localise around the lighter particles leading to an onion-like arrangement of the different ion species in the crystal [5] (Fig. 3a). In the present experiments, the central core of the crystals is formed by the laser-cooled Ca^+ ions.

The shell-like ion arrangements strongly affect the ion dynamics during ejection into the TOF-MS flight tube and lead to a dispersion of the time-of-flight of the ions depending on their initial position in the crystal. The extended ion packets impinging on the detector give rise to bimodal distributions in the TOF spectrum with maxima at two distinct arrival times [6]. The widths of the bimodal TOF signals strongly depend on the amount of lighter ions in the original Coulomb crystal which determine the diameter of the shells of heavier ions.

To illustrate this effect, MD simulations of Coulomb crystals were performed which are composed of two ion species with mass 40 u and 92 u. The number of ions with mass 92 u was fixed to 100 and the number of ions of mass 40 u was varied between 100 and 500. The electrode potentials in the simulations were chosen to be identical to the ones in the experiment. Simulated TOF mass spectra of these crystals are presented in Fig. 3b. While the lighter species produces a single peak in the TOF trace, the heavier ions show a bimodal distribution the width of which increases with the number of light ions.

In order to assign masses to the different bands observed in the product TOF-MS spectrum of Fig. 3 of the main text, MD simulations of Coulomb crystals consisting of Ca^+ ions (mass 40 u) and different compositions of molecular ions corresponding to the reactants and products were performed. A comparison of the experimental data with a best-fit simulation is shown in Fig. 4. The corresponding ion composition of the Coulomb crystal assumed in this simulation is detailed in Tab. 1. For clarity, the simulated TOF signals are shown in different colours to highlight the contribution from each mass. A global time offset of $\lesssim 0.5$ μs was added to the simulated spectrum in order to match the

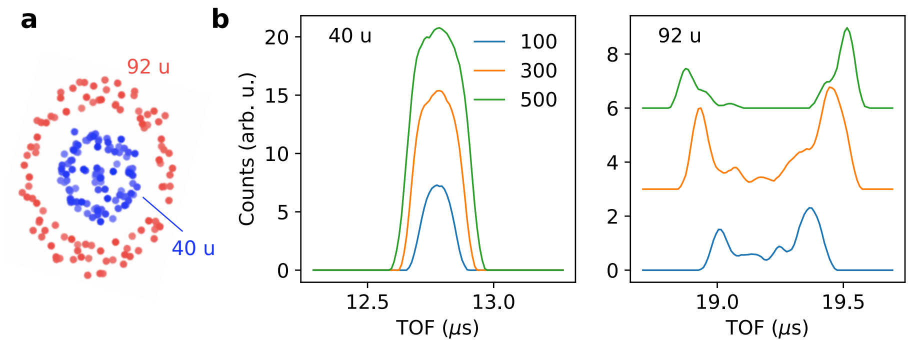

Supplementary Figure 3: **Influence of Coulomb-crystal size on ion time-of-flight spectra**. **a** Simulated Coulomb crystal consisting of ions with mass of 40 u (blue) and mass 92 u (red). **b** Simulated TOF mass spectra for Coulomb crystals consisting of a variable number (100, 300, 500) of ions with mass 40 u and 100 ions of mass 92 u. With increasing number of lighter ions, the width of the bimodal time-of-flight distribution for the ions with mass 92 u increases. TOF traces for the species at mass 92 u are vertically offset for clarity.

position of the Ca^+ peak in the experimental data. We find an overall satisfactory agreement between experiment and simulation. In particular, the observed splittings of the individual mass signals are reproduced in the simulated TOF spectra. Small differences in the splittings and positions of the peaks in the simulation are attributed to different sizes and compositions of the Coulomb crystal in the experiment and simulations and to the possible presence of additional heavy ion species which are not accounted for in the simulations. Because of these uncertainties, we estimate the accuracy of the determination of the masses to be ± 1 u.

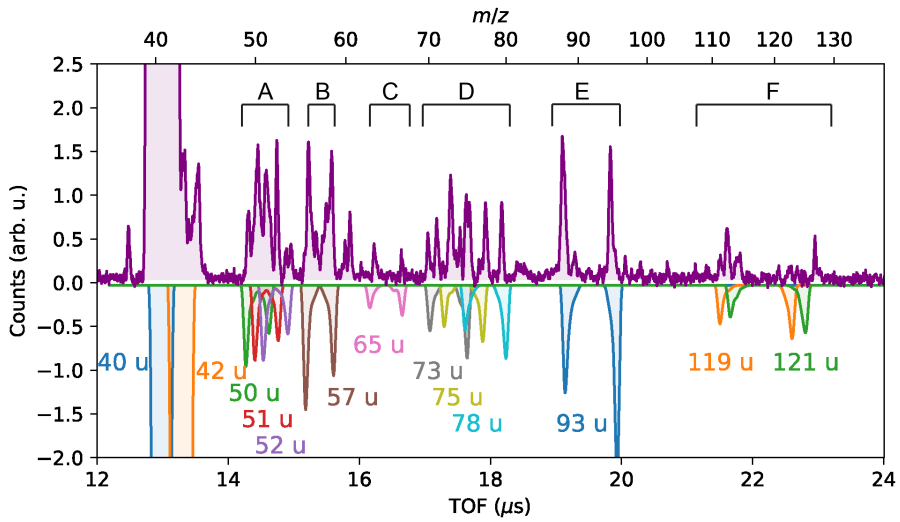

Supplementary Figure 4: **Experimental and simulated TOF mass spectra.** The experimental TOF mass spectrum of Fig. 3 in the main text (top trace) is compared with a simulation (lower inverted trace). Features corresponding to different masses are highlighted in different colours for clarity. The estimated uncertainty of the mass determination is ± 1 u, see text for discussion.

Based on the agreement between experiment and simulation, we assign the bands A–F in Fig. 3 of the main text to different molecular formulae as summarised in table 2.

Ion mass (u)	Number of ions	Ion mass (u)	Number of ions
40	450	73	20
42	210	75	16
50	20	78	20
51	20	93	48
52	20	119	16
57	36	121	16
65	10		

Supplementary Table 1: **Ion composition of Coulomb crystal** for the simulated TOF mass spectrum of Fig. 4.

Label	Assigned masses (u)	Possible compounds
A	50,51,52	$C_4H_n^+$, n=2,3,4
B	57	$CaOH^+$
C	65	$C_5H_5^+$
D	77,78	$C_6H_n^+$, $n = 5, 6$
E	93	$C_7H_9^+$
F	119, 121	$CaBr^+$

Supplementary Table 2: **Assignment of the main TOF bands originating from the reaction of DBB with propene ions** (not accounting for masses produced by background reactions). The estimated uncertainty of the mass determination is ±1 u, see text for discussion.

2.2 Product fragmentation pathways

The fragmentation of unstable products of radical cation reactions in the gas phase is a widely observed phenomenon, see, e.g., Refs. [7–12]. Considering the excess energy of > 60 kcal/mol of the present reaction and the constant presence of near-infrared and near-ultraviolet laser light in the experiment, a range of different pathways leading from the DA cycloadduct to the observed fragments listed in Tab. 2 are conceivable. It is plausible that in a first step, a cyclic $C_7H_{10}^+$ moiety (m/z = 94 u) is formed through the loss of the two bromine atoms from the DA cycloadduct, the 2-dibromo-4-methyl-cyclohexene radical cation (Fig. 1a). This fragment ion can further undergo hydrogen loss forming protonated toluene (93 u, band E in Fig. 3 of the main text). Elimination of a methyl group or methane can result in the benzene (78 u) and further H loss in the phenyl (77 u) ion (band D) [13–15]. As an alternative, a sequence of hydrogen losses, possibly involving a skeletal rearrangement to the tropylium ion [16], and the elimination of C_2H_2 results in the formation of the cyclopentadienyl cation $C_5H_5^+$ (65 u, band C)[12]. The $C_4H_n^+$ fragments (band A) could partially result from further breakup of this C_5 moiety, but are mostly accounted for by background reactions of DBB with Ca^+ (see main text).

In order to achieve a more detailed understanding of the fragmentation pathways, further studies at a higher mass resolution which would enable the unambiguous determination of the fragment masses in combination with theoretical calculations of the potential energy surfaces would be required. However, we note the striking general resemblance of the fragmentation pattern of the product of the reaction of DBB with propene ions shown in Fig. 4 (and Fig. 3 of the main text) with the ones observed in the mass spectra of cyclic compounds such as toluene [12]. This similarity provides further evidence that the product of the title reaction is indeed the cyclic DA adduct. However, it cannot be ruled out that some fragmentation (especially loss of Br) occurs already before ring formation. A more

comprehensive exploration of the potential energy surface of the system would be needed to clarify this possibility.

Supplementary Note 3 Potential energy surface

Supplementary Table 3: Single-point energies (SPE) of stationary points on the PES of the reaction system and $\langle S^2 \rangle$ values calculated at the M06-2X/def2-TZVPP level of theory with Stuttgart ECP including relativistic effects on the bromine atoms [17]. Refer to Fig. 4 of the main text for the nomenclature of the structures.

Structure	SPE (kcal/mol)	$\langle S^2 \rangle$
gauche	0.00	-
s-trans	-0.50	-
$I1^g$	-12.40	0.756
$I1^t$	-12.97	0.756
$I2^g$	-20.18	0.783
$I2^t$	-25.16	0.790
$I3^g$	-10.72	0.757
$P1^g$	-58.75	0.753
$P1^t$	-31.72	0.777
$P2^g$	-62.40	0.753
$P2^t$	-33.29	0.778
$P3^g$	-31.90	0.780
$P3^t$	-32.24	0.780
$TS1^g$	-9.91	0.758
$TS1^t$	-12.40	0.757
$TS2^g$	-17.91	0.781
$TS2^t$	-22.49	0.789
$TS3^g$	-10.11	0.756
$TS4^g$	-18.34	0.786
$TS4^t$	-23.18	0.790
$TS5^g$	-14.11	0.784
$TS5^t$	-18.52	0.790
$TS6^g$	-58.55	0.753
$TS6^t$	-31.61	0.777
TS7	-24.48	0.7596
TS^{iso}	-16.30	0.7752

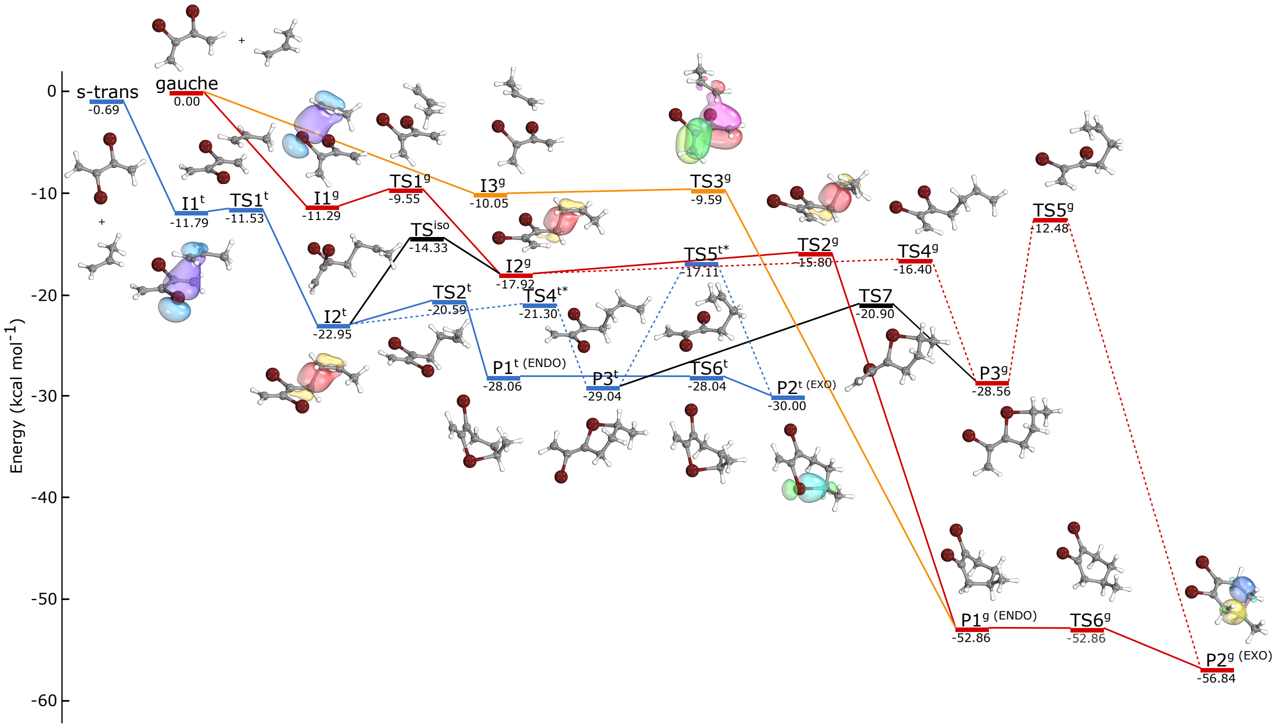

Supplementary Figure 5: **Detailed potential energy surface of the title reaction** supplementing Fig. 4 of the main text. The figure shows additional conformational pathways including the *exo*-form of the DA cycloadduct, as well as a path leading to the formation of a five-membered ring in the *s-trans*-path (isomers P1^{t}, P2^{t} and P3^{t}) which can isomerise to the DA cycloadduct via TS7.

Supplementary Note 4 Adiabatic capture theory

Rotationally adiabatic-capture rate constants were calculated using the theory developed by Clary and co-workers [18, 19]. The long-range ion-molecule interaction potential adapted in the calculations included the charge-induced dipole and charge-permanent dipole terms:

$$V(R,\beta) = -\frac{\alpha q^2}{2R^4} - \frac{q\mu_D \cos\beta}{R^2}, \tag{2}$$

with q the ion charge, α the molecular isotropic polarisability, μ_D the permanent dipole moment of the neutral molecule and μ the reduced mass of the collision partners. The ion-molecule distance is denoted R and the orientation angle of the molecular dipole relative to the ion-molecule axis is denoted β. The molecular constants for DBB used in these calculations were taken from Ref. [20]. A set of rotationally adiabatic, centrifugally corrected potential energy curves $V_{JjK_aK_c\Omega}(R)$ was calculated (Figure 5a and b of the main text), where J denotes the quantum number of total angular momentum of the system, Ω the quantum number of its projection on the intermolecular axis and R the intermolecular distance. It is assumed that all collisions up to a maximum angular momentum J_{max} for which the collision energy E_{col} exceeds the centrifugal barrier of the effective potential lead to a successful reactive encounter. The reaction cross sections were calculated as [19]

$$\sigma(j, K_a, K_c, E_c) = \frac{\pi\hbar^2}{2\mu E_c}\frac{1}{2j+1}\sum_{\Omega=-j}^{j}(J_{\max}(j, K_a, K_c, \Omega)+1)^2. \tag{3}$$

The reaction-rate constants were then obtained as $k(j, K_a, K_c, E_c) = \sigma(j, K_a, K_c, E_c)v$ where v denotes the collision velocity.

Supplementary References

[1] Schmid, P. C., Greenberg, J., Miller, M. I., Loeffler, K. & Lewandowski, H. J. An ion trap time-of-flight mass spectrometer with high mass resolution for cold trapped ion experiments. *Rev. Sci. Instrum* **88**, 123107 (2017).

[2] Hankin, S. M., Villeneuve, D. M., Corkum, P. B. & Rayner, D. M. Intense-field laser ionization rates in atoms and molecules. *Phys. Rev. A* **64**, 013405 (2001).

[3] Wiese, J., Olivieri, J.-F., Trabattoni, A., Trippel, S. & Küpper, J. Strong-field photoelectron momentum imaging of OCS at finely resolved incident intensities. *New J. Phys* **21**, 083011 (2019).

[4] Manura, D. & Dahl, D. *SIMION 8.0/8.1 User manual.* Scientific Instrument Services, Inc., Ringoes, NJ, rev. 5 edn. (2011).

[5] Willitsch, S. Coulomb-crystallised molecular ions in traps: methods, applications, prospects. *Int. Rev. Phys. Chem.* **31**, 175–199 (2012).

[6] Rösch, D., Gao, H., Kilaj, A. & Willitsch, S. Design and characterization of a linear quadrupole ion trap for high-resolution coulomb-crystal time-of-flight mass spectrometry. *EPJ Tech. Instrum.* **3**, 5 (2016).

[7] Eberlin, M. N. Gas-phase polar cycloadditions. *Int. J. Mass Spectrom.* **235**, 263–278 (2004).

[8] Goebbert, D. J., Liu, X. & Wenthold, P. G. Reactions of diacetylene radical cation with ethylene. *J. Am. Soc. Mass Spectrom.* **15**, 114–120 (2004).

[9] Colorado, A., Barket, J. D., Hurst, M. J. & Shepson, B. P. A fast-response method for determination of atmospheric isoprene using quadrupole ion trap mass spectrometry. *Anal. Chem.* **70**, 5129–5135 (1998).

[10] Hofmann, M. & Schaefer, H. F. Pathways for the reaction of the butadiene radical cation $C_4H_6^{+}$, with ethylene. *J. Phys. Chem. A* **103**, 8895–8905 (1999).

[11] Bouchoux, G., Salpin, J.-Y. & Turecek, F. Cycloaddition reactions between 1,3-butadiene radical cations and ethene in the gas phase. *Rapid Commun. Mass Spectrom* **8**, 325–328 (1994).

[12] Gross, J. *Mass Spectrometry* (Springer International Publishing, Cham, 2017), 3 edn.

[13] Kuck, D., Schneider, J. & Grützmacher, H.-F. A study of gaseous benzenium and toluenium ions generated from 1,4- dihydro- and 1-methyl-1,4-dihydro-benzoic acids. *J. Chem. Soc., Perkin Trans. 2* 689–696 (1985).

[14] Schröder, D., Schwarz, H., Milko, P. & Roithová, J. Dissociation routes of protonated toluene probed by infrared spectroscopy in the gas phase. *J. Phys. Chem. A* **110**, 8346–8353 (2006).

[15] Wang, Z.-C. *et al.* The gas-phase methylation of benzene and toluene. *Int. J. Mass Spectrom.* **429**, 6–13 (2018).

[16] Lifshitz, C. Tropylium ion formation from toluene: Solution of an old problem in organic mass spectrometry. *Acc. Chem. Res.* **27**, 138–144 (1994).

[17] Bergner, A., Dolg, M., Küchle, W., Stoll, H. & Preuß, H. Ab initio energy-adjusted pseudopotentials for elements of groups 13–17. *Mol. Phys.* **80**, 1431–1441 (1993).

[18] Clary, D. Rate constants for the reactions of ions with dipolar polyatomic molecules. *J. Chem. Soc., Faraday Trans. 2* **83**, 139–148 (1987).

[19] Stoecklin, T., Clary, D. C. & Palma, A. Rate constant calculations for ion-symmetric top and ion-asymmetric top reactions. *J. Chem. Soc. Faraday Trans.* **88**, 901 (1992).

[20] Kilaj, A. *et al.* Quantum-chemistry-aided identification, synthesis and experimental validation of model systems for conformationally controlled reaction studies: Separation of the conformers of 2,3-dibromobuta-1,3-diene in the gas phase. *Phys. Chem. Chem. Phys.* **22**, 13431–13439 (2020).
